# Draft Genome Sequence of Desulfosporosinus fructosivorans Strain 63.6F^T^, Isolated from Marine Sediment in the Baltic Sea

**DOI:** 10.1128/MRA.00427-19

**Published:** 2019-08-01

**Authors:** Bela Hausmann, Verona Vandieken, Petra Pjevac, Katharina Schreck, Craig W. Herbold, Alexander Loy

**Affiliations:** a Centre for Microbiology and Environmental Systems Science, Department of Microbiology and Ecosystem Science, Division of Microbial Ecology, University of Vienna, Vienna, Austria; b Joint Microbiome Facility of the Medical University of Vienna and the University of Vienna, Vienna, Austria; c Department of Laboratory Medicine, Medical University of Vienna, Vienna, Austria; d Paleomicrobiology Group, Institute for Chemistry and Biology of the Marine Environment (ICBM), University of Oldenburg, Oldenburg, Germany; e Austrian Polar Research Institute, Vienna, Austria; University of Maryland School of Medicine

## Abstract

Desulfosporosinus fructosivorans strain 63.6F^T^ is a strictly anaerobic, spore-forming, sulfate-reducing bacterium isolated from marine sediment in the Baltic Sea. Here, we report the draft genome sequence of D. fructosivorans 63.6F^T^.

## ANNOUNCEMENT

The genus Desulfosporosinus contains sulfate-reducing, endospore-forming bacteria with versatile substrate utilization capabilities (1–4). Desulfosporosinus fructosivorans strain 63.6F^T^ was isolated from the subsurface of marine sediment at Landsort Deep in the Baltic Sea, recovered during IODP Expedition 347 (5). Strain 63.6F^T^ grows with sulfate as an electron acceptor and fructose, lactate, and ethanol as electron donors, but it does not grow with pyruvate, methanol, and butanol as electron donors (5).

*D. fructosivorans* strain 63.6F^T^ was cultivated as described previously (5). Genomic DNA was isolated using the DNeasy PowerSoil DNA isolation kit (Mo Bio), and sequencing libraries were prepared using the Nextera XT kit (Illumina) and sequenced with the Illumina HiSeq 2000 platform, yielding 35 million 120-bp paired-end reads. The reads were inspected with FastQC (version 0.11.5, http://www.bioinformatics.babraham.ac.uk/projects/fastqc/) and quality trimmed at a Phred quality score of 10 using the BBDuk function of BBMap (version 34.94, https://sourceforge.net/projects/bbmap/). Quality-trimmed reads were assembled using SPAdes (version 3.6.2) (6) and subsequently iteratively (*n* = 4) reassembled with SPAdes (version 3.11.1), using contigs of >1 kb from the previous assembly as the “trusted contigs” input and iterating kmers from 11 to 121 in steps of 10. The draft genome sequence consists of 38 scaffolds with a total size of 6,122,266 bp, a G+C content of 42.3%, and an *N*_50_ value of 405,172 bp. Based on CheckM (7), the completeness of the draft genome is 99.0%, with 15 duplicated single-copy marker genes.

Taxonomic placement of *D. fructosivorans* strain 63.6F^T^ was verified using a concatenated alignment of 18 unique marker genes using the GTDB toolkit (8) and IQ-TREE (9). The most similar genome was that of Desulfosporosinus sp. strain palsa_1189 (GenBank accession number GCA_003132105), with an average nucleotide identity (ANI) of 83% (alignment fraction, 0.61) (10), well below the intraspecies threshold of 96.5% (11). Average amino acid identities (AAIs) to published *Desulfosporosinus* genomes ranged from 72% to 81% (filtered for alignment fraction, >0.6) (12).

The genome was annotated using RAST (13) and the NCBI Prokaryotic Genome Annotation Pipeline. It contains 5,842 coding sequences (CDS), 7 rRNAs, 58 tRNAs, and 4 noncoding RNAs (ncRNAs). Like other members of the sulfate-reducing genus *Desulfosporosinus*, *D. fructosivorans* has genes involved in the canonical pathway for dissimilatory sulfate reduction, including those for sulfate adenylyltransferase (*sat*), adenylyl-sulfate reductase (*aprBA*), and dissimilatory sulfite reductase (*dsrAB*). Trimeric sulfite reductase encoding genes (*asrABC*) were not detected (14). The name-giving ability of fructose degradation is possible via the phosphotransferase system.

### Data availability.

This whole-genome shotgun project has been deposited at DDBJ/ENA/GenBank under the accession numbers PRJNA529082 and SPQQ00000000. The version described in this paper is SPQQ01000000.
